# Ultrasound as the Primary Predictor of Perioperative Hemorrhage in Low-to-Moderate Risk Placenta Accreta Spectrum: A Prospective Comparison with MRI in Women with Placenta Previa

**DOI:** 10.3390/diagnostics16131960

**Published:** 2026-06-24

**Authors:** Sul Lee, Hojun Lee, Hyun-Joo Lee, Eun-Hee Yu, Jong-Kil Joo, Seung-Chul Kim

**Affiliations:** 1 Department of Obstetrics and Gynecology, Pusan National University School of Medicine, 179 Gudeok-Ro, Seo-gu, Busan 49241, Republic of Korea; ch9061@naver.com (S.L.); atouchofbreeze@gmail.com (H.-J.L.); hoyyeh@naver.com (E.-H.Y.); jongkilj@hanmail.net (J.-K.J.); 2 Biomedical Research Institute, Pusan National University Hospital, 179 Gudeok-ro, Seo-gu, Busan 49241, Republic of Korea; hojun8702@naver.com; 3 Department of Radiology, Pusan National University Hospital, 179 Gudeok-ro, Seo-gu, Busan 49241, Republic of Korea

**Keywords:** placenta accreta spectrum, placenta previa, ultrasound, magnetic resonance imaging, perioperative hemorrhage, postpartum hemorrhage, prenatal diagnosis, inter-modality agreement

## Abstract

**Background/Objectives:** Placenta accreta spectrum (PAS) is an increasingly prevalent and potentially life-threatening complication in women with placenta previa. Despite widespread clinical use, the inter-modality agreement between prenatal ultrasound and MRI and their comparative value for predicting perioperative hemorrhage remain poorly characterized, particularly in low-to-moderate risk populations where placenta accreta predominates. We aimed to compare inter-modality agreement between standardized ultrasound and MRI impressions and to evaluate each modality’s predictive value for perioperative hemorrhage. **Methods:** This prospective cohort study enrolled 47 women with placenta previa who underwent both standardized ultrasound and MRI prospectively between 28 + 0 and 32 + 6 weeks of gestation, with perioperative outcomes collected at the time of cesarean delivery. Both modalities were classified using a three-tier impression system (None/Suspected/Likely) based on standardized structural, vascular, and invasive marker composites. The primary outcome was inter-modality agreement (linearly weighted Cohen’s κ); secondary outcomes were the association of each modality’s impression with postpartum hemorrhage (PPH; estimated blood loss ≥ 1000 mL) and estimated blood loss (EBL). **Results:** PAS was confirmed in 18 of 47 women (38.3%), predominantly placenta accreta (83.3%). Inter-modality agreement was fair (weighted κ = 0.263), structural concordance was moderate (κ = 0.539), while vascular agreement was near-absent (κ = 0.085). Ultrasound impression demonstrated a dose-dependent association with PPH rates (38.5%, 52.9%, and 82.4% across None, Suspected, and Likely tiers; *p* = 0.048) and EBL (800, 1000, and 1800 mL; *p* = 0.003), with logistic regression confirming a 2.70-fold increase in PPH odds per tier (*p* = 0.018; AUC 0.657). MRI impression was not associated with PPH (*p* = 1.000), EBL (*p* = 0.743), or PAS status (*p* = 0.741; AUC 0.543). Serum AFP was significantly elevated in women with PPH (*p* = 0.005). **Conclusions:** In this accreta-predominant, low-to-moderate risk cohort, ultrasound—but not MRI—demonstrated a significant dose-dependent association with perioperative hemorrhage. These findings should not be interpreted as evidence of general MRI inadequacy but rather as reflecting the specific imaging context in which MRI’s strengths in deep invasion characterization are less clinically determinative. These results support ultrasound as the primary tool for hemorrhage risk stratification in this population.

## 1. Introduction

Placenta previa, defined as the location of the placenta overlying or adjacent to the internal cervical os, complicates approximately 0.3–0.5% of all pregnancies and remains a leading cause of postpartum hemorrhage and peripartum maternal morbidity worldwide [1]. The incidence of placenta previa has risen substantially over recent decades, largely attributable to increasing rates of cesarean delivery, uterine instrumentation, and assisted reproductive technology [2,3]. Of particular concern is the co-occurrence of placenta accreta spectrum (PAS) disorder, an umbrella term encompassing abnormal placental adherence (accreta), myometrial invasion (increta), and parametrial or adjacent organ invasion (percreta). Among women with placenta previa, the risk of PAS increases substantially with the number of prior uterine surgeries, reaching up to 40% in those with two or more previous cesarean deliveries [4].

PAS disorder is associated with life-threatening peripartum hemorrhage, with reported mean estimated blood loss often exceeding 3000 mL and frequently necessitating massive transfusion [5]. Hysterectomy remains the definitive treatment in severe cases, with rates approaching 80–90% in women with percreta [4]. Beyond immediate hemorrhagic complications, PAS contributes significantly to maternal intensive care unit admission, urologic injury, and maternal mortality, rendering it one of the most formidable challenges in contemporary obstetrics [5]. Accurate antenatal identification of PAS is therefore critical to enable multidisciplinary planning, optimized surgical timing, and allocation of appropriate resources prior to delivery [6].

Grayscale and color Doppler ultrasound is the recommended first-line imaging modality for antenatal evaluation of PAS in women with placenta previa, endorsed by major guidelines including those of the International Federation of Gynecology and Obstetrics (FIGO) and the American College of Obstetricians and Gynecologists (ACOG) [6,7]. Established sonographic markers have been classified into structural features (loss of the retroplacental clear space, myometrial thinning), vascular features (intraplacental lacunae, high-velocity turbulent lacunar flow), and invasive features (uterine-bladder interface disruption, bridging vessels) [8]. Meta-analyses have reported pooled sensitivity and specificity of ultrasound for PAS of approximately 91% and 97%, respectively; however, performance varies considerably across studies, and ultrasound may be limited by operator experience, maternal body habitus, and posterior placentation [9].

Magnetic resonance imaging (MRI) has emerged as an adjunct modality, particularly when ultrasound findings are equivocal or when posterior placental location limits sonographic assessment [10]. MRI-specific markers of PAS include T2-weighted dark intraplacental bands, heterogeneous placental signal, uterine bulging, abnormal placental vascularity, myometrial disruption, and bladder invasion [11]. Although several studies have evaluated the diagnostic performance of MRI for PAS, direct prospective comparisons with ultrasound using standardized marker definitions remain limited. Furthermore, most existing studies have been retrospective and performed at variable gestational ages and have not systematically assessed inter-modality agreement at the level of individual marker categories [12]. Critically, few studies have examined how specific imaging markers—whether structural, vascular, or invasive—correlate with actual perioperative hemorrhage, the most clinically consequential outcome of PAS [13].

The optimal gestational window of 28–32 weeks has been proposed for PAS imaging assessment, as placental invasion becomes more reliably identifiable while allowing sufficient time for multidisciplinary delivery planning [7]. We therefore conducted a prospective cohort study of women with placenta previa who underwent both standardized ultrasound and MRI at 28–32 weeks of gestation. The objectives of this study were threefold: (1) to evaluate and compare the diagnostic accuracy of ultrasound and MRI for PAS against a surgical reference standard; (2) to assess the overall and category-specific agreement between ultrasound and MRI markers, classified as structural, vascular, and invasive; and (3) to determine the correlation between imaging-derived markers and perioperative hemorrhage, including estimated blood loss and postpartum hemorrhage requiring intervention.

## 2. Materials and Methods

### 2.1. Study Design and Setting

This prospective cohort study was conducted at the Department of Obstetrics and Gynecology, Pusan National University Hospital, a tertiary referral center for high-risk obstetrics. The study protocol was approved by the Institutional Review Board of Pusan National University Hospital (IRB No. 2402-003-135), and written informed consent was obtained from all participants prior to enrollment.

### 2.2. Participants

Women with a confirmed diagnosis of placenta previa were eligible for enrollment. Placenta previa was defined as placental tissue overlying or within 2 cm of the internal cervical os on transvaginal ultrasound. Inclusion criteria were (1) singleton pregnancy; (2) placenta previa confirmed at ≥28 weeks of gestation; and (3) completion of both standardized ultrasound and MRI examinations between 28 + 0 and 32 + 6 weeks of gestation. Exclusion criteria were: (1) multiple gestation; (2) major fetal structural anomaly; (3) technically inadequate imaging (non-diagnostic image quality on either modality); and (4) delivery or pregnancy loss prior to imaging completion.

### 2.3. Ultrasound Examination

All ultrasound examinations were performed between 28 + 0 and 32 + 6 weeks of gestation using a high-resolution Voluson E10 system (GE Healthcare, Kretztechnik, Zipf, Austria). Examinations were performed with a partially filled maternal bladder using a transabdominal approach, supplemented by transvaginal imaging when transabdominal views were suboptimal.

The following grayscale and color Doppler markers were systematically evaluated and recorded as present or absent, classified into three categories [8]:

Structural markers:

- Loss of the retroplacental clear space (absence of the hypoechoic zone between the placenta and myometrium);
- Myometrial thinning (myometrial thickness < 1 mm or indiscernible).

Vascular markers:

- Intraplacental lacunae (irregular vascular spaces within the placenta, grade ≥ 2);
- High-velocity turbulent flow within lacunae on color Doppler.

Invasive markers:

- Uterine–bladder interface disruption (interruption of the hyperechoic line between the bladder wall and uterine serosa);
- Bridging vessels (vessels extending from the placenta across the uterine serosa toward the bladder or adjacent structures on color Doppler).

Marker definitions followed the consensus criteria established by the Society for Maternal-Fetal Medicine Placenta Accreta Spectrum Ultrasound Marker Task Force [8]. Individual marker findings were recorded, and a composite overall impression was assigned using the following three-tier classification: none (no features suggestive of PAS), suspected (one or more equivocal features present without definitive invasive signs), or likely (two or more definitive markers present in at least one category, or any single high-specificity finding such as uterine–bladder interface disruption or bridging vessels).

### 2.4. MRI Examination

MRI was performed within 7 days of the ultrasound examination, between 28 + 0 and 32 + 6 weeks of gestation, using a 3.0-Tesla system (Skyra; Siemens Healthineers, Erlangen, Germany) without gadolinium contrast administration. All examinations were conducted with the patient in the supine position. The protocol included T2-weighted turbo spin-echo (TSE) sequences in three orthogonal planes (TR/TE 3610/84 ms; FOV 380 × 304 mm; slice thickness 6 mm), T2-weighted half-Fourier single-shot turbo spin-echo (HASTE) sequences in three orthogonal planes (TR/TE 800/80 ms; FOV 350 × 284 mm; slice thickness 7 mm), a T1-weighted sequence, and diffusion-weighted imaging (DWI).

The following MRI markers were systematically evaluated and recorded as present or absent, classified into the same three categories as the ultrasound [11]:

Structural markers:

- Thinning or loss of the retroplacental T2 dark zone (absence or disruption of the low-signal myometrial band on T2-weighted images);
- Myometrial thinning (myometrial thickness < 1 mm or indiscernible);
- Focal disruption of the myometrium (full-thickness myometrial defect on T2-weighted images).

Vascular markers:

- Dark intraplacental bands on T2-weighted images (linear or curvilinear hypointense bands within the placenta);
- Heterogeneous placental signal intensity (non-homogeneous T2 signal);
- Abnormal or disorganized placental vascularity (irregular or prominent placental vessels).

Invasive markers:

- Placental bulging (focal outward protrusion of the uterine contour);
- Lumpy contour and rounded edge of the uterus (lobulated or irregular uterine margin);
- Bladder or adjacent structural invasion (loss of T2 hypointense bladder wall or direct organ involvement);
- Focal exophytic placental mass (placental tissue extending beyond the uterine serosa).

The overall MRI impression was classified using the same three-tier scale of none, suspected, or likely, applying identical definitions to those used for the ultrasound.

### 2.5. Image Interpretation

Ultrasound and MRI examinations were interpreted independently by dedicated readers blinded to the findings of the opposing modality and to intraoperative outcomes. Ultrasound images were reviewed by a single maternal-fetal medicine specialist (an assistant professor with 6 years of postgraduate experience in obstetric ultrasound). MRI examinations were reviewed by a single radiologist (a clinical assistant professor with 3 years of postgraduate experience). Both readers were provided with the patient’s gestational age, placental location, and prior obstetric history (number of previous cesarean deliveries) but were blinded to each other’s interpretations and to the surgical outcome at the time of image review.

### 2.6. Reference Standard

The reference standard for PAS diagnosis was the intraoperative surgical assessment recorded by the attending obstetrician at the time of delivery. PAS was graded as grade 0 (no PAS; placenta separated without evidence of abnormal adherence), grade 1 (accreta; abnormal placental adherence without myometrial invasion), grade 2 (increta; myometrial invasion), or grade 3 (percreta; invasion of the uterine serosa or adjacent organs), in accordance with the FIGO clinical classification for PAS disorders [14]. In cases where cesarean hysterectomy was performed, the surgical diagnosis was confirmed by histopathological examination of the uterine specimen. For the primary diagnostic accuracy analysis, grades 1–3 were combined and classified as PAS-positive, and grade 0 as PAS-negative.

### 2.7. Outcome Measures

The primary outcome was the overall inter-modality agreement between ultrasound and MRI for PAS diagnosis, assessed using the three-tier composite impression scale (none/suspected/likely).

EBL was quantified using a standardized approach combining suction canister volume measurement and gravimetric weighing of surgical sponges (post-use weight minus dry weight) and was recorded prospectively at the time of cesarean delivery by the attending surgical team. Secondary outcomes were (1) diagnostic accuracy of ultrasound and MRI individually against the surgical reference standard (sensitivity, specificity, PPV, NPV, and AUC); (2) category-specific inter-modality agreement between ultrasound and MRI within each marker category (structural, vascular, and invasive), using composite binary category scores (category-positive if any marker within the category was present); (3) correlation between imaging findings and estimated blood loss (EBL); and (4) ability of imaging findings to predict postpartum hemorrhage (PPH), defined as EBL ≥ 1000 mL at cesarean delivery, consistent with the WHO criteria [15].

The post-delivery clinical parameters recorded included estimated blood loss (EBL), shock index (heart rate divided by systolic blood pressure), units of packed red blood cells (pRBC) transfused, and hysterectomy.

### 2.8. Statistical Analysis

Continuous variables are reported as mean ± standard deviation or median (interquartile range) according to the distribution, and categorical variables as frequencies and percentages. Normality was assessed using the Shapiro–Wilk test.

Diagnostic accuracy. Sensitivity, specificity, positive predictive value (PPV), negative predictive value (NPV), and the area under the receiver operating characteristic curve (AUC) with 95% confidence intervals were calculated for ultrasound and MRI individually against the surgical reference standard. Two cut-points were evaluated: ≥suspected (suspected or likely classified as PAS-positive) and likely only (only likely classified as PAS-positive). The comparison of AUCs between ultrasound and MRI was performed using the DeLong method.

Inter-modality agreement. Overall agreement between ultrasound and MRI impression (none/suspected/likely) was quantified using linearly weighted Cohen’s kappa (κ) with 95% confidence intervals. Agreement was interpreted as poor (κ < 0.20), fair (0.21–0.40), moderate (0.41–0.60), substantial (0.61–0.80), or almost perfect (>0.80). Category-specific agreement (structural, vascular, and invasive composite binary scores) was assessed using unweighted Cohen’s kappa.

Hemorrhage correlation. The association between imaging impression scores (0 = none, 1 = suspected, 2 = likely) and continuous EBL was evaluated using Spearman’s rank correlation coefficient (ρ). Category-specific Spearman correlations were computed for each composite category score against EBL. Differences in EBL across the three imaging impression groups were assessed using the Kruskal–Wallis test with post hoc pairwise comparisons using the Mann–Whitney U test with Bonferroni correction. EBL was additionally compared between PAS-positive and PAS-negative groups using the Mann–Whitney U test.

PPH prediction. Binary logistic regression was used to assess the association between imaging findings and PPH (EBL ≥ 1000 mL). Univariable analyses were performed for the overall imaging impression and each category composite score. Variables with *p* < 0.10 on univariable analysis were entered into multivariable models, and the results are reported as odds ratios (ORs) with 95% confidence intervals.

Sample size. Sample size was calculated based on the primary outcome of weighted kappa for overall US-MRI agreement. Assuming an expected κ of 0.70, a null hypothesis κ of 0.40, and a two-sided α of 0.05 and power of 0.80, a minimum of 38 participants were required. Accounting for an estimated 15% dropout rate due to delivery prior to imaging completion or technically inadequate images, a target enrollment of 45 women was set.

All statistical analyses were performed using IBM SPSS Statistics, version 26.0 (IBM Corp., Armonk, NY, USA). A two-sided *p*-value < 0.05 was considered statistically significant.

## 3. Results

### 3.1. Study Population

A total of 47 women with placenta previa who underwent both standardized ultrasound and MRI examinations between 28 + 0 and 32 + 6 weeks of gestation were included. Eighteen women (38.3%) were confirmed to have PAS at the time of cesarean delivery, of whom 15 (83.3%) had placenta accreta and three (16.7%) had placenta increta; no cases of placenta percreta were identified. The remaining 29 women (61.7%) showed no evidence of abnormal placental invasion. Baseline characteristics are summarized in Table 1.

The two groups were well matched in demographics. Maternal age (median 34 vs. 35 years; *p* = 0.860), BMI (*p* = 0.991), and gravidity (*p* = 0.788) were similar between PAS-positive and PAS-negative women; parity was modestly higher in the PAS-positive group (median 0.5 vs. 0; *p* = 0.019), the only demographic variable that reached statistical significance. Established risk factors for PAS did not differ significantly between groups: myomectomy (5.6% vs. 3.4%; *p* = 1.000), prior cesarean delivery (33.3% vs. 20.7%; *p* = 0.493), prior endometrial procedures (16.7% vs. 17.2%; *p* = 1.000), and use of assisted reproductive technology (22.2% vs. 27.6%; *p* = 0.744) were all comparable. Gestational age at delivery (*p* = 0.875) and serum AFP (MoM; median 1.2 vs. 1.1; *p* = 0.185) were also similar between groups.

Placental characteristics were also comparable between groups. Posterior placentation was the predominant location in both groups (55.6% in PAS-positive vs. 72.4% in PAS-negative; *p* = 0.488), and complete previa was the most common previa type overall, accounting for 77.8% and 65.5% of the PAS-positive and PAS-negative groups, respectively (*p* = 0.287).

Perioperative outcomes differed markedly between groups. EBL was significantly greater in the PAS-positive group (median 1650 [IQR 1200–2375] mL vs. 800 [500–1000] mL; *p* < 0.001), and PPH (EBL ≥ 1000 mL) occurred in 17 of 18 PAS-positive women (94.4%) compared with 11 of 29 PAS-negative women (37.9%; *p* < 0.001). Packed red blood cell transfusion was required in 94.4% of PAS-positive versus 55.2% of PAS-negative women (*p* = 0.007). Hysterectomy was required in five patients, all within the PAS-positive group (27.8% vs. 0%; *p* = 0.006).

### 3.2. Imaging Impressions and Diagnostic Accuracy

**Figure 1:**
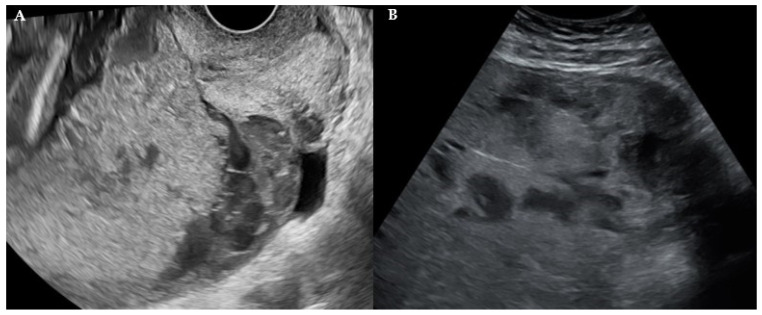
Representative ultrasound findings suggestive of placenta accreta spectrum in women with placenta previa. (**A**) Transvaginal grayscale ultrasound demonstrating loss of the retroplacental clear zone with multiple irregular intraplacental lacunae. (**B**) Transabdominal grayscale ultrasound demonstrating absence of the retroplacental clear space with heterogeneous placental echotexture at the lower uterine segment.

**Figure 2:**
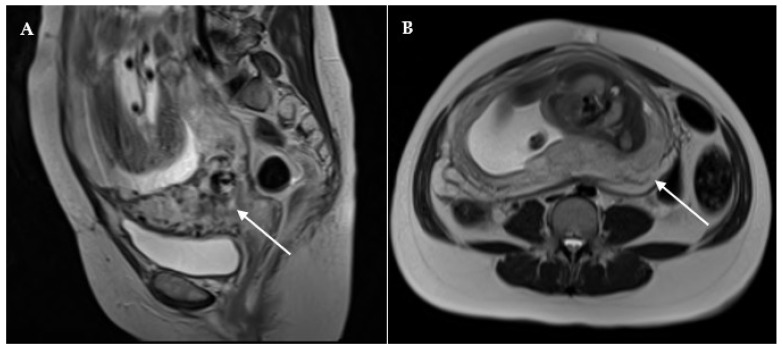
Representative magnetic resonance imaging findings suggestive of placenta accreta spectrum in women with placenta previa. (**A**) Sagittal T2-weighted TSE image demonstrating multiple intraplacental T2-hypointense dark bands (white arrow) extending through the placental substance. (**B**) Axial T2-weighted HASTE image demonstrating focal disruption of the utero-placental interface (white arrow) with loss of the normal low-signal myometrial junctional zone and irregular placental contour, suggestive of placental invasion.

Applying the three-tier classification (None/Suspected/Likely), ultrasound categorized 13 women (27.7%) as None, 17 (36.2%) as Suspected, and 17 (36.2%) as Likely. MRI yielded a shifted distribution, classifying 14 women (29.8%) as None, 12 (25.5%) as Suspected, and 21 (44.7%) as Likely. The diagnostic accuracy of individual ultrasound and MRI markers for PAS detection is presented in Table 2. Among individual ultrasound markers, placental lacunae demonstrated the highest sensitivity (72.2%; 95% CI 59.4–85.0%), while loss of retroplacental clear space had the best overall discriminatory performance (AUC 0.702). Myometrial thinning and utero-bladder interface disruption each achieved high specificity (89.7%) with modest sensitivity. Among the MRI markers, myometrial thinning had the highest sensitivity (55.6%) and AUC (0.640); bladder invasion and exophytic mass were absent in all 47 patients. Using the “Likely” tier as a positive cut-off for PAS diagnosis, ultrasound achieved a sensitivity of 55.6%, specificity of 75.9%, and PPV of 58.8% (AUC 0.657). Corresponding MRI values were 50.0% sensitivity, 58.6% specificity, and 42.9% PPV (AUC 0.543). When the ≥suspected threshold was applied (suspected or likely classified as PAS-positive), ultrasound sensitivity increased to 83.3% but specificity declined sharply to 34.5% (PPV 44.1%, NPV 76.9%, AUC 0.589); 15 of 18 PAS-positive cases were captured, but 19 of 29 PAS-negative women were also classified as positive. Under the same threshold, MRI achieved a sensitivity of 77.8% and specificity of 34.5% (PPV 42.4%, NPV 71.4%, AUC 0.561). At both thresholds, ultrasound demonstrated higher overall discriminatory performance than MRI. Representative ultrasound and MRI findings from this cohort are illustrated in Figure 1 and Figure 2, respectively.

Reference standard: intraoperative surgical findings (PAS + n = 18, PAS − n = 29).

Composite category: positive if any constituent marker present. Bladder invasion and exophytic mass absent in all patients.

AUC = (sensitivity + specificity)/2.

Abbreviations: AUC, area under the receiver operating characteristic curve; MRI, magnetic resonance imaging; NPV, negative predictive value; PAS, placenta accreta spectrum; PPV, positive predictive value.

## 3.3. Primary Outcome: Inter-Modality Agreement

The cross-tabulation of ultrasound and MRI impression tiers and category-specific agreement statistics are presented in Table 3. The overall unweighted agreement between ultrasound and MRI three-tier impressions was 44.7% (21 of 47 patients). The linearly weighted Cohen’s kappa was 0.263 (95% CI −0.037 to 0.563), indicating fair agreement. Category-specific binary kappa values were markedly heterogeneous: structural features showed moderate agreement (κ = 0.539; 95% CI 0.300–0.777; *p* < 0.001), while vascular (κ = 0.085; 95% CI −0.183 to 0.352; *p* = 0.535) and invasive (κ = 0.132; 95% CI −0.249 to 0.513; *p* = 0.497) categories showed poor agreement. Notably, among the 17 ultrasound-Suspected cases, eight (47.1%) were classified as MRI-None, reflecting the largest single source of inter-modality discordance. Overall unweighted agreement: 44.7% (21/47). Linearly weighted Cohen’s κ = 0.263 (95% CI −0.037 to 0.563); fair agreement.

## 3.4. Secondary Outcomes: Association with Perioperative Hemorrhage

PPH rates demonstrated a significant dose-dependent increase across ascending ultrasound impression tiers—38.5%, 52.9%, and 82.4% for None, Suspected, and Likely, respectively (*p* = 0.048)—while EBL increased correspondingly from 800 [IQR 600–1000] to 1000 [600–1200] to 1800 [1000–2500] mL (Kruskal–Wallis *p* = 0.003; Table 4). Post hoc pairwise analysis (Bonferroni-corrected) confirmed significant differences between the None and Likely tiers (*p* = 0.011) and the Suspected and Likely tiers (*p* = 0.014). On logistic regression, each one-tier increase in ultrasound impression was associated with a 2.70-fold increase in the odds of PPH (95% CI 1.19–6.13; *p* = 0.018; Table 5). Spearman correlation between ultrasound impression score and continuous EBL was ρ = 0.481 (*p* < 0.001; Table 5). The ultrasound structural category was the strongest individual predictor of both EBL (ρ = 0.489; *p* < 0.001) and PPH (OR 6.15; 95% CI 1.46–25.96; *p* = 0.013; Table 5).

In contrast, MRI impression showed no significant association with PPH (*p* = 1.000), EBL (Kruskal–Wallis *p* = 0.743; Table 4), or Spearman correlation with EBL (ρ = 0.098; *p* = 0.511; Table 5) and did not predict PPH on logistic regression (OR 1.11 per tier; 95% CI 0.56–2.19; *p* = 0.772; Table 5). Median EBL was 1000 mL across all three MRI impression tiers. The MRI structural category showed a modest Spearman correlation with EBL (ρ = 0.338; *p* = 0.020) but did not independently predict PPH (*p* = 0.068).

Neither ultrasound nor MRI impression was significantly associated with PAS status across tiers (ultrasound *p* = 0.105; MRI *p* = 0.741).

## 3.5. Exploratory Analysis

Serum AFP (MoM) was significantly higher in women who experienced PPH than in those who did not (median 1.242 [IQR 1.066–1.526] vs. 0.952 [0.870–1.184]; *p* = 0.005), whereas AFP did not differ significantly between PAS-positive and PAS-negative women (*p* = 0.185). Prior surgical history, including previous cesarean delivery, myomectomy, and endometrial procedure, was not significantly associated with PAS status or PPH (all *p* > 0.35).

## 4. Discussion

In this prospective cohort of women with placenta previa undergoing standardized prenatal imaging, ultrasound and MRI three-tier impressions demonstrated only fair inter-modality agreement (weighted κ = 0.263). Crucially, only the ultrasound impression exhibited a significant dose-dependent association with perioperative hemorrhage; MRI impression showed no association with either PPH or EBL across any analytical approach. A defining feature of this cohort—the predominance of grade 1 accreta (83.3% of PAS-positive cases) with an absence of bladder invasion or exophytic mass—may specifically amplify the divergence in predictive value between the two modalities, a distinction not well represented in the literature.

### 4.1. Inter-Modality Agreement

The fair but imperfect agreement observed between ultrasound and MRI (overall weighted κ = 0.263) indicates that the two modalities frequently assign different risk tiers to the same patient. Category-specific analysis revealed a particularly striking divergence: structural features showed moderate binary agreement (κ = 0.539), whereas vascular and invasive domains demonstrated near-zero kappa values (0.085 and 0.132, respectively). The vascular discordance was directionally asymmetric: ultrasound identified vascular abnormalities in 32 of 47 patients (68.1%), compared with 19 of 47 (40.4%) on MRI. This asymmetry is consistent with the technical superiority of color Doppler ultrasound in detecting low-velocity intraplacental flow within lacunar spaces, which the T2-weighted MRI may underrepresent in the absence of gadolinium contrast [6,7]. The largest single source of discordance was reclassification of ultrasound-Suspected cases as MRI-None (8 of 17 cases, 47.1%), suggesting that MRI most frequently “down-grades” equivocal ultrasound findings, rather than providing independent risk elevation. Prior comparative data have reported broadly similar diagnostic AUCs for the two modalities at the population level [12]; our findings extend this by demonstrating that concordant population-level performance does not preclude substantial individual-level disagreement, a clinically important distinction when the two studies conflict in practice.

### 4.2. Ultrasound Impression and Hemorrhage Prediction

The dose-dependent relationship between ultrasound impression tier and both PPH rate and EBL was among the most clinically relevant findings of this study. Women classified as ultrasound-Likely experienced a median EBL of 1800 mL and an 82.4% PPH rate, compared with 800 mL and 38.5% in the ultrasound-None group—differences that remained significant after Bonferroni correction and that were replicated by Spearman correlation (ρ = 0.481) and logistic regression (OR 2.70 per tier; *p* = 0.018). Among individual category scores, the structural composite—comprising loss of retroplacental clear space and myometrial thinning—was the strongest predictor of both continuous EBL (ρ = 0.489) and PPH (OR 6.15; *p* = 0.013), suggesting that disruption of the myometrial–placental interface provides hemorrhage-relevant information even in the absence of frank transmural invasion. These findings are consistent with prior work demonstrating that specific sonographic features predict intraoperative blood loss in PAS [13] and extend those observations to a standardized composite impression framework.

The relationship between diagnostic threshold and clinical utility also warrants consideration. Lowering the positive criterion to ≥suspected increased ultrasound sensitivity markedly (55.6% → 83.3%), reducing missed PAS cases from eight to three; however, specificity declined to 34.5%, with 19 of 29 PAS-negative women classified as positive (PPV 44.1%). The net effect was a decrease in overall discriminatory performance (AUC 0.589 vs. 0.657 at the likely threshold), indicating that the sensitivity gain was outweighed by a disproportionate loss of specificity—a finding that reflects the low PAS prevalence within the Suspected tier (5 of 17 cases, 29.4%). MRI showed limited discrimination at both thresholds (AUC 0.543 and 0.561 for likely-only and ≥suspected, respectively). These findings suggest that the likely cutoff is preferable when a high-specificity result is needed to guide intensive perioperative resource allocation, whereas a ≥suspected criterion may be appropriate in screening contexts where minimizing missed PAS cases takes priority—accepting a substantially higher false-positive burden. We acknowledge that PAS status and ultrasound impression are correlated and that a formal mediation analysis to disentangle the direct and PAS-mediated components of the ultrasound-PPH association was not feasible in this sample. The observed association should therefore be interpreted as reflecting the hemorrhage-stratifying information contained in the ultrasound impression framework as a whole rather than as evidence of a pathway entirely independent of PAS pathophysiology.

### 4.3. MRI and the Accreta-Predominant Cohort

In contrast to ultrasound, MRI impression was entirely non-predictive of hemorrhage, with median EBL fixed at 1000 mL across all three impression tiers—a striking flatness that persisted across logistic (OR 1.11; *p* = 0.772) and correlational (ρ = 0.098; *p* = 0.511) analyses. We propose that this finding is substantially explained by the grade composition of our cohort. Placenta accreta—superficial trophoblastic invasion of the myometrium without complete transmural penetration—accounted for 83.3% of PAS-positive cases, and none of the patients had bladder invasion or exophytic mass. The imaging signs most specifically captured by MRI, including T2 dark bands through the full myometrial thickness, bladder wall irregularity, and exophytic placental protrusion, reflect the deep myometrial or extramyometrial invasion that characterizes placenta increta or percreta [14]. When these high-grade features are largely absent, MRI’s incremental contribution to hemorrhage stratification may be intrinsically limited.

Ultrasound, by contrast, is uniquely sensitive to the intraplacental vascular alterations that typify even low-grade accreta. Placental lacunae—irregular hypoechoic spaces reflecting abnormal vascular lakes—were present on ultrasound in 68.1% of the cohort, more than three times the prevalence of the vascular abnormalities detected by MRI (40.4%). These lacunar spaces represent the pathological vascular architecture of abnormal placentation and appear to reflect the hemorrhagic burden at delivery, even when invasion depth is limited to placenta accreta. This interpretation is consistent with published data showing that the presence and turbulence grade of placental lacunae correlate with intraoperative blood loss in mixed-grade PAS cohorts [13] and extends that relationship to predominantly accreta populations. In cohorts with higher proportions of increta and percreta, where deep myometrial or parametrial penetration drives catastrophic hemorrhage, MRI’s capacity to delineate invasion extent may well provide greater additive value—an important caveat to the generalizability of our findings.

The observation that MRI impression was non-predictive of PAS status itself (*p* = 0.741)—with similar PAS rates across the None, Suspected, and Likely tiers—further underscores that the composite MRI impression, as derived in this accreta-predominant sample, lacked meaningful discriminatory stratification. Rekawek et al. reported that MRI altered clinical management in 36.6% of referred PAS cases, though incorrect diagnostic changes were nearly as frequent as correct ones [10]; our data suggest that, in low-to-moderate-risk previa patients with predominantly superficial invasion, MRI-based reclassification may be particularly prone to introducing imprecision rather than resolving it. It is important to emphasize that the non-predictive performance of MRI observed in this study should not be interpreted as evidence of general MRI inadequacy in PAS evaluation. Rather, our findings reflect the specific imaging context of an accreta-predominant cohort in which (1) MRI features most characteristic of high-grade PAS—T2 dark bands through the full myometrial thickness, bladder wall irregularity, and exophytic placental mass—were largely absent; (2) gadolinium contrast, which may enhance detection of placental vascularity and invasion depth, was not administered; and (3) the distribution of invasion grades was heavily weighted toward superficial accreta. In cohorts with higher proportions of increta and percreta, or when gadolinium-enhanced MRI is systematically employed, MRI may well provide substantially greater incremental value for both diagnosis and hemorrhage stratification.

### 4.4. AFP and Surgical History

Serum AFP was included as a secondary variable in this study given established evidence linking elevated second-trimester maternal serum AFP (MS-AFP) to abnormal placentation. Kupferminc et al. [16] first reported that MSAFP elevation occurred in 45% of women with PAS compared with none of the controls (*p* < 0.001), concluding that unexplained MSAFP elevation should heighten clinical suspicion for placenta accreta and associated hemorrhage. Oztas et al. [17] subsequently demonstrated, in a cohort of 316 women with complete placenta previa, that elevated MS-AFP was the sole statistically significant serum predictor of morbidly adherent placenta requiring hysterectomy (1.25 MoM cutoff: sensitivity 85.9%, specificity 71.4%; OR 25.3). A recent systematic scoping review of 29 studies confirmed that all seven cohorts examining second-trimester AFP found elevated levels in women with PAS at delivery, yielding a pooled odds ratio of 9.73 (95% CI 2.25–42.10) [18].

In the present cohort, AFP did not differ significantly between PAS-positive and PAS-negative women (*p* = 0.185). This may reflect the accreta-predominant composition of our cohort, as the AFP–PAS association in prior studies appears most pronounced in cases with deeper myometrial invasion, where greater disruption of the placental barrier drives AFP leakage into the maternal circulation. Notably, however, AFP was significantly higher in women who experienced PPH than in those who did not (median 1.242 [IQR 1.066–1.526] vs. 0.952 [0.870–1.184] MoM; *p* = 0.005), suggesting that elevated AFP may reflect a degree of placental vascular disruption that predisposes to hemorrhage through a pathway at least partially independent of histological invasion grade. Whether AFP provides clinically useful additive information for hemorrhage risk stratification in previa patients warrants prospective evaluation in larger cohorts.

Prior surgical history—including previous cesarean delivery, myomectomy, and endometrial procedures—was not significantly associated with either PAS status or PPH (all *p* > 0.35), likely reflecting inadequate statistical power given the modest sample size.

### 4.5. Limitations

This study has several limitations. First, the modest sample size (N = 47, PAS-positive n = 18) substantially limits the precision of all estimated effect sizes, as reflected in the wide confidence intervals throughout (e.g., OR 2.70, 95% CI 1.19–6.13 for ultrasound impression predicting PPH). These findings should be regarded as hypothesis-generating rather than confirmatory, and multicenter replication in larger, grade-stratified cohorts is required before the results can be considered generalizable. Second, the reference standard for PAS diagnosis relied on intraoperative surgical assessment in the majority of cases. Histopathological confirmation was available only for the five patients (27.8% of PAS-positive) who underwent hysterectomy. In the remaining 13 PAS-positive patients, diagnosis rested on the surgeon’s intraoperative assessment of abnormal placental adherence, which is susceptible to inter-observer variability and grade misclassification, particularly at the accreta–increta boundary. Non-differential misclassification of this type would tend to attenuate observed associations, suggesting that the true associations between imaging impression and hemorrhage outcomes may be modestly stronger than reported. Third, the three-tier impression framework applied here was analytically derived from marker composites rather than based on a validated prospective classification and may not align precisely with other published scoring systems. Fourth, both ultrasound and MRI examinations were interpreted by single experienced readers at each modality, and interobserver reliability was not assessed. The reproducibility of the three-tier impression classification—particularly for vascular and invasive markers, where subjective judgment plays a greater role—therefore remains uncertain. Prospective validation studies involving multiple independent readers would be needed to establish the inter-rater reliability of this classification framework before clinical implementation. Finally, MRI was performed without gadolinium contrast, which may have reduced sensitivity to placental vascularity; whether dynamic contrast-enhanced MRI improves hemorrhage prediction—particularly in the accreta-predominant setting—remains to be investigated.

## 5. Conclusions

This prospective study demonstrates fair inter-modality agreement between ultrasound and MRI in women with placenta previa and identifies ultrasound impression as the sole imaging predictor of perioperative hemorrhage. In an accreta-predominant cohort reflecting a clinically common low-to-moderate PAS risk profile, ultrasound sensitivity to intraplacental vascular abnormalities appeared to drive its predictive value, whereas MRI showed limited hemorrhage discrimination. These findings support ultrasound as the primary modality for hemorrhage risk stratification in this population and underscore the need for grade-stratified, multicenter studies to define the specific clinical contexts in which MRI provides incremental diagnostic value.

## Figures and Tables

**Table 1:** Baseline characteristics.

Variable	All (N = 47)	PAS-Positive (n = 18)	PAS-Negative (n = 29)	*p*-Value
Demographics				
Age, years	34 [32.5–36]	34 [33–36]	35 [32–36]	0.860
BMI, kg/m^2^	20.7 [19.5–22.1]	20.5 [19.9–21.6]	20.8 [19.1–22.2]	0.991
Gravidity	2 [1–3]	1.5 [1–2.8]	2 [1–3]	0.788
Parity	0 [0–1]	0.5 [0–1]	0 [0–0]	**0.019**
Obstetric history				
Myomectomy, n (%)	2 (4.3%)	1 (5.6%)	1 (3.4%)	1.000
Prior C/S, n (%)	12 (25.5%)	6 (33.3%)	6 (20.7%)	0.493
1 previous	9 (19.1%)	4 (22.2%)	5 (17.2%)	0.716
2 previous	3 (6.4%)	2 (11.1%)	1 (3.4%)	0.549
Endometrial procedure, n (%)	8 (17.0%)	3 (16.7%)	5 (17.2%)	1.000
ART, n (%)	12 (25.5%)	4 (22.2%)	8 (27.6%)	0.744
Current pregnancy				
AFP, MoM	1.1 [1.0–1.4]	1.2 [1.0–1.5]	1.1 [0.9–1.3]	0.185
GA at delivery, weeks	37 [36–37]	37 [36–37]	37 [36–37]	0.875
Placenta location				0.488
Anterior	14 (29.8%)	7 (38.9%)	7 (24.1%)	
Posterior	31 (66.0%)	10 (55.6%)	21 (72.4%)	
Other	2 (4.3%)	1 (5.6%)	1 (3.4%)	
Placenta previa type				0.287
Complete	33 (70.2%)	14 (77.8%)	19 (65.5%)	
Partial	7 (14.9%)	3 (16.7%)	4 (13.8%)	
Marginal	2 (4.3%)	1 (5.6%)	1 (3.4%)	
Low-lying	5 (10.6%)	0 (0.0%)	5 (17.2%)	
Delivery and perioperative outcomes				
Delivery mode				0.726
Planned CS	36 (76.6%)	13 (72.2%)	23 (79.3%)	
Emergency CS	11 (23.4%)	5 (27.8%)	6 (20.7%)	
EBL, mL	1000 [750–1450]	1650 [1200–2375]	800 [500–1000]	**<0.001**
PPH (EBL ≥ 1000 mL), n (%)	28 (59.6%)	17 (94.4%)	11 (37.9%)	**<0.001**
Post-bleeding shock index	1.0 [0.9–1.2]	1.2 [1.0–1.2]	1.0 [0.9–1.1]	**0.027**
Packed RBC transfusion, n (%)	33 (70.2%)	17 (94.4%)	16 (55.2%)	**0.007**
Hysterectomy, n (%)	5 (10.6%)	5 (27.8%)	0 (0.0%)	**0.006**

Data presented as median [IQR] or n (%). Bold *p*-values indicate statistical significance (*p* < 0.05). Low-lying: includes cases with placenta extending to but not covering the internal os. Mann–Whitney U test for continuous variables; Fisher’s exact test for categorical variables. Abbreviations: ART, assisted reproductive technology; BMI, body mass index; C/S, cesarean section; CS, cesarean section; EBL, estimated blood loss; GA, gestational age; IQR, interquartile range; MoM, multiples of the median; PAS, placenta accreta spectrum; PPH, postpartum hemorrhage (EBL ≥ 1000 mL).

**Table 2:** Diagnostic accuracy of individual ultrasound and MRI markers for PAS detection.

Marker	Sensitivity, % (95% CI)	Specificity, % (95% CI)	PPV, %	NPV, %	AUC
Ultrasound					
Loss of retroplacental clear space	61.1 (47.2–75.0)	79.3 (67.7–90.9)	61.1	73.3	0.702
Myometrial thinning (<1 mm)	33.3 (19.9–46.8)	89.7 (80.9–98.4)	75.0	62.5	0.615
Lacunae (any grade)	72.2 (59.4–85.0)	34.5 (20.9–48.1)	41.9	64.7	0.534
High-turbulence lacunae	38.9 (25.0–52.8)	79.3 (67.7–90.9)	58.3	63.9	0.591
Utero-bladder interface disruption	16.7 (6.0–27.3)	89.7 (80.9–98.4)	75.0	62.2	0.532
Bridging vessels	16.7 (6.0–27.3)	89.7 (80.9–98.4)	75.0	62.2	0.532
Composite categories					
Structural	61.1 (47.2–75.0)	75.9 (63.6–88.1)	57.9	77.4	0.685
Vascular	72.2 (59.4–85.0)	34.5 (20.9–48.1)	41.9	64.7	0.534
Invasive	16.7 (6.0–27.3)	86.2 (76.3–96.1)	60.0	61.0	0.514
MRI					
T2 dark zone/myometrial thinning	22.2 (10.3–34.1)	75.9 (63.6–88.1)	40.0	62.7	0.490
Myometrial thinning (MRI)	55.6 (41.3–69.8)	72.4 (59.6–85.2)	55.6	72.4	0.640
Focal myometrial disruption	5.6 (0.0–12.1)	79.3 (67.7–90.9)	16.7	58.7	0.424
Dark intraplacental bands	33.3 (19.9–46.8)	69.0 (55.7–82.2)	40.0	62.5	0.511
Heterogeneous placenta	22.2 (10.3–34.1)	82.8 (72.0–93.6)	44.4	62.6	0.525
Abnormal vascularity	11.1 (2.1–20.1)	96.6 (91.3–100.0)	66.7	60.4	0.538
Placental bulging	22.2 (10.3–34.1)	82.8 (72.0–93.6)	44.4	62.6	0.525
Lumpy uterine contour	27.8 (15.0–40.6)	86.2 (76.3–96.1)	55.6	64.2	0.570
Bladder invasion	0.0	100.0	—	61.7	0.500
Exophytic mass	0.0	100.0	—	61.7	0.500
Composite categories					
Structural	61.1 (47.2–75.0)	51.7 (37.4–66.0)	43.8	68.2	0.564
Vascular	44.4 (30.2–58.7)	62.1 (48.2–75.9)	44.4	62.1	0.533
Invasive	33.3 (19.9–46.8)	75.9 (63.6–88.1)	46.2	64.7	0.546

**Table 3:** Inter-modality agreement between ultrasound and MRI: (**A**) Three-tier impression cross-tabulation (US rows × MRI columns); (**B**) Category-level binary agreement.

(**A**)					
**US\MRI**	**None (n = 14)**	**Suspected (n = 12)**	**Likely (n = 21)**	**Total**	
None (n = 13)	5	4	4	13	
Suspected (n = 17)	8	4	5	17	
Likely (n = 17)	1	4	12	17	
Total	14	12	21	47	
(**B**)					
**Category**	**US Positive, n**	**MRI Positive, n**	**Agreement, %**	**κ (95% CI)**	***p*-Value**
Structural	18	25	76.6%	0.539 (0.300–0.777)	<0.001
Vascular	32	19	51.1%	0.085 (−0.183 to 0.352)	0.535
Invasive	7	13	70.2%	0.132 (−0.249 to 0.513)	0.497

Category positive = any individual marker within that category being present. Kappa interpreted as <0.20 slight, 0.21–0.40 fair, 0.41–0.60 moderate, 0.61–0.80 substantial, and >0.80 almost perfect. Abbreviations: MRI, magnetic resonance imaging; US, ultrasound; κ, Cohen’s kappa.

**Table 4:** Three-tier impression by modality: PAS rate, PPH rate, and EBL.

Modality	Impression	n	PAS-Positive	PPH ^†^	EBL, mL (Median [IQR]) ^‡^
Ultrasound	None	13	3 (23.1%)	5 (38.5%)	800 [600–1000]
MRI	None	14	4 (28.6%)	8 (57.1%)	1000 [650–1200]

Post hoc pairwise EBL (Bonferroni-corrected, US only): None vs. Likely *p* = 0.011; Suspected vs. Likely *p* = 0.014; None vs. Suspected *p* = 1.000. All MRI pairwise comparisons *p* = 1.000. Logistic regression (PPH, per one-tier increase): US OR 2.70 (95% CI 1.19–6.13), *p* = 0.018; MRI OR 1.11 (95% CI 0.56–2.19), *p* = 0.772. † Chi-square test; ‡ Kruskal–Wallis test. Abbreviations: EBL, estimated blood loss; IQR, interquartile range; MRI, magnetic resonance imaging; PAS, placenta accreta spectrum; PPH, postpartum hemorrhage (EBL ≥ 1000 mL); US, ultrasound.

**Table 5:** Association between imaging scores and perioperative hemorrhage.

Imaging Score	Spearman ρ (vs. EBL)	*p*-Value	OR for PPH (95% CI)	*p*-Value
Ultrasound				
Structural category	0.489	<0.001	6.15 (1.46–25.96)	**0.013**
Vascular category	0.234	0.114	NS	—
Invasive category	0.180	0.227	NS	—
Overall impression (0–2)	0.481	<0.001	2.70 (1.19–6.13)	**0.018**
MRI				
Structural category	0.338	0.020	NS	—
Vascular category	−0.137	0.360	NS	—
Invasive category	0.028	0.851	NS	—
Overall impression (0–2)	0.098	0.511	NS	—
PAS status	0.703	<0.001	27.82 (3.23–239.24)	**0.002**

OR presented for variables with *p* < 0.05 on univariable logistic regression. NS, not significant (*p* > 0.05). —, not applicable. Bold *p*-values indicate *p* < 0.05. Abbreviations: EBL, estimated blood loss; MRI, magnetic resonance imaging; NS, not significant; OR, odds ratio; PAS, placenta accreta spectrum; PPH, postpartum hemorrhage (EBL ≥ 1000 mL).

## Data Availability

The data that support the findings of this study are available from the corresponding author upon reasonable request.
